# Linking a Population Biobank with National Health Registries—The Estonian Experience

**DOI:** 10.3390/jpm5020096

**Published:** 2015-04-16

**Authors:** Liis Leitsalu, Helene Alavere, Mari-Liis Tammesoo, Erkki Leego, Andres Metspalu

**Affiliations:** 1 Estonian Genome Center, University of Tartu, Riia 23b, Tartu 51010, Estonia; E-Mails: liis.leitsalu@ut.ee (L.L.); helene.alavere@ut.ee (H.A.); mari-liis.tammesoo@ut.ee (M.-L.T.); 2 Institute of Molecular and Cell Biology, University of Tartu, Riia 23, Tartu 51010, Estonia; 3 Hansson, Leego & Partner, Turu 1, 51014 Tartu, Estonia; E-Mail: erkki.leego@hlp.ee

**Keywords:** biobanking, electronic health records, personalized medicine, Estonian Biobank, re-contacting, linking, registries, databases

## Abstract

The Estonian population-based biobank, with 52,000 participants’ genetic and health data, is the largest epidemiological cohort in the Baltic region. Participants were recruited through a network of medical professionals throughout Estonia (population 1.34 million). Unique legislation as well as a broad consent form give the Estonian Genome Center, a research institute of the University of Tartu, permission to re-contact participants and to retrieve participants’ data from national registries and databases. In addition to two re-contacting projects to update the health data of participants, extensive clinical characterizations have been retrieved from national registries and hospital databases regularly since 2010. Acquiring data from electronic health records and registries has provided a means to update and enhance the database of the Genome Center in a timely manner and at low cost. The resulting database allows a wide spectrum of genomic and epidemiological research to be conducted with the aim of benefitting public health. Future plans include linking the genome center database with the national health information system through X-road and exchanging data in real time, as well as using the genetic data and the technical infrastructure available for piloting personalized medicine in Estonia.

## 1. From a Biobank to Personalized Medicine

### 1.1. Objectives

In the advent of personalized (precision) medicine, the linking of biobanks with the electronic medical records and health databases is the most crucial step. The Estonian Human Genes Research Act stipulated that the results of the research conducted with data available in the Estonian Biobank must be used to improve public health. Therefore, we have investigated the situation in Estonia, where the nation wide health database of the Estonian National Health Information System (ENHIS) and other more specific registries are available in respect to improving the phenotypic content of the biobank database. The vision for the future is that the ENHIS database and molecular profiling data of the patients will be used to calculate disease risk and likely drug response. Since both databases (ENHIS database and the Estonian Biobank database) are continually improving, estimates of the disease risk(s) and probable drug response must be re-calculated regularly. This can be done automatically and at a large scale only when these two databases are connected in real time. This paper describes some of the steps taken towards this goal and issues met during the process.

### 1.2. Brief History

The Estonian Biobank was established over a decade ago as one the first population based biobanks [1]. The goals were to collect health and genetic data for the population and conduct genetic research to benefit public health.

The initial aim was to recruit participants until the longitudinal and prospective cohort reached 52,000 participants (gene donors), which constitutes about 5 percent of the adult population of Estonia. This makes the Estonian Genome Center of the University of Tartu (EGCUT) cohort the largest epidemiological cohort not just in Estonia but in the whole Baltic region. More recent efforts started to concentrate on using the available genome-wide data together with the phenotypic data in research projects. Simultaneously, new projects were launched to update and enhance the phenotypic data of the participants either through re-contacting the participants or via national registries and databases. While genomic discovery efforts continue, the EGCUT is now actively looking for ways to translate research results into medical practice.

## 2. Biobanking and Legislation

### HGRA and Consent Form

Prior to the establishment of the Estonian Biobank a legal framework was created to specifically regulate the activities related to the biobank. The Estonian Human Genes Research Act (HGRA) [2] was passed by the Estonian Parliament in 2000 to regulate the establishment and maintenance of the Estonian Biobank. The objective of the HGRA is also to regulate research and biobank-related activities of the EGCUT. Additionally, the HGRA protects the rights of the participants (gene donors). As required by the HGRA, all participants joining the biobank have signed an informed consent form [3] to ensure voluntary and informed participation.

Several aspects covered by the HGRA are crucial for the development of the EGCUT database as well as the application of the database in a variety of research projects:

- While the funding for research projects conducted by the EGCUT is competitively based, the specimen collection stored in the biobank and the database itself is maintained by the state as stated in the HGRA. This might contribute to the reasons that the biobank is strongly supported by the public as shown by annual telephone polls over the past decade [1].
- Tissue samples, genetic information and the description of health (phenotypic data) all have a unique code and are anonymous. However, decoding is permitted for specific purposes described by the HGRA. These include renewal of the description of health or in order to contact participants. Decoding the data allows health descriptions to be linked with the genetic data of a participant, which is essential for genome-based health research.
- The HGRA permits the participants to be re-contacted in order to renew and supplement the data available at the EGCUT. Re-contacting also makes it possible to obtain new tissue samples either when the first sample has been destroyed or does not contain sufficient DNA or when other types of tissue samples are necessary for specific projects (epigenetics, expression studies).
- The act permits additional information to be received from other databases to supplement the description of the state of health of the participants.

Additionally, the consent form includes aspects that are crucial for the development of the EGCUT database as well as for the application of the database in a variety of research projects:

- The Gene Donor Consent Form signed by all participants is a “broad consent”. This type of consent requests permission to use the samples collected for future research purposes in general (with the objectives to study the links between genes, environmental factors and lifestyle with physical characteristics, health and disease), without specifically identifying each of the projects. This makes it possible to conduct a wide range of research, which would have been difficult if not impossible to foresee at the time of participant recruitment. Such research projects are only conducted after approval from the Research Ethics Committee of the University of Tartu.
- The broad consent form also asks for permission to receive additional information from other databases. Linking with other databases and registries makes it possible to renew and enhance the database of the EGCUT on a regular basis without having to re-contact the participants.
- The participants have the right to be aware of their data available in the database and they have the right to receive genetic counseling upon accessing that data. In other words, the participants have the right to have the research results returned. The participants can also grant their physician access to that information. This makes it possible to conduct research projects where personal genome information is introduced into medical practice.

## 3. Building, Maintaining, and Enhancing the Database

### 3.1. Study Design

The participants were recruited by medical professionals as thoroughly described in Leitsalu *et al.* [1]. For that purpose, a network of primary care medical professionals from all 15 Estonian counties was established with over half of the registered family physicians in Estonia involved (454 general practitioners). In addition, special recruitment cabinets were set up in regional hospitals and at the EGCUT. The recruitment visit involved a health examination, collection of a blood sample, and completion of the electronic EGCUT questionnaire on the health and lifestyle of the participant. The broad network of medical professionals helped to recruit participants throughout the country creating a cohort of 52,000 participants by the end of 2010. This cohort represents about 5 percent of the adult population and is a good representation of the general adult population of Estonia [1].

### 3.2. Data Collected

The data available in the EGCUT database can be roughly divided into phenotypic data collected and genotypic data generated based on the type of information, but also divided into baseline and follow-up based on the time of data collection.

**Figure 1:**
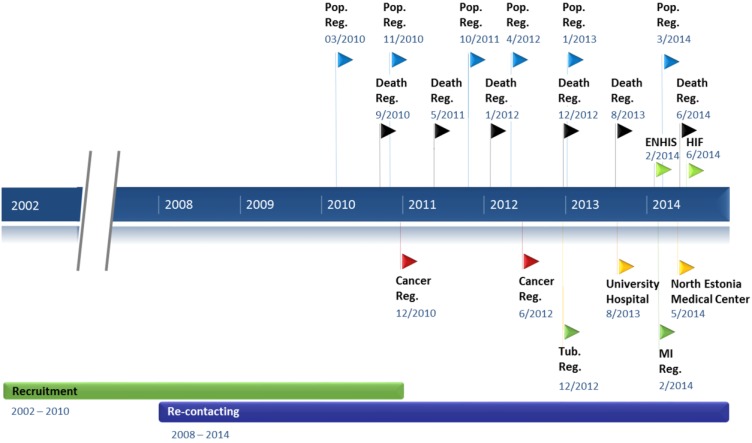
Timeline of the data collection procedures. The timeline showing the recruitment period, re-contacting projects, and frequency and timing of linking with registries and databases. Recruitment—Baseline data collection at the recruitment visit; Re-contacting—2nd timepoint data collection in re-contacting projects; Pop. Reg.—Population Register; Death Reg.—Estonian Causes of Death Registry; ENHIS—Estonian National Health Information System; HIF—Estonian Health Insurance Fund; Cancer Reg.—Estonian Cancer Registry; Tub. Reg.—Estonian Tuberculosis Registry; University Hospital—Database of the Tartu University Hospital; North Estonia Medical Center—Database of the North Estonia Medical Centre; MI Reg.—Myocardial Infarction Registry.

At recruitment, a thorough computer-assisted questionnaire consisting of approximately 330 questions and over 1000 data fields was filled out by the recruiter (Figure 1) [4]. It contained questions of a personal and demographic nature as well as questions about genealogy, health behavior, chronotype and diseases. The fact that the recruitment of participants was carried out by medical professionals ensured the reliability of anthropometric measurements and also meant that when documenting the medical history and medication use, official health records could be used, in addition to the information reported by the participant. For the purpose of validation, the source of the information was recorded (self-reported or supported by medical records).

A blood sample was taken from all participants from which DNA, white blood cells, and plasma were isolated and stored in the biobank. Genotypic or sequence data is being gradually generated for research purposes. As of March 2015, genome-wide array data was available for over 21,700 participants, and approximately 3000 exome sequences and 2000 whole-genome sequences, 1000 mRNA expression arrays, and 1000 RNA sequences have been generated.

### 3.3. Re-Contacting Participants

The biobank is most valuable with the extensive health data accompanying the genetic data. In addition to the health information collected at baseline, the information is continuously updated through re-contacting projects as well as through linking with electronic databases as permitted by the HGRA. Re-contacting has been carried out in two waves in 2008–2010 and in 2011–2014 with close to 2000 participants successfully re-examined (Figure 1). The response rate in re-contacting projects was between 57.2 percent and 41.1 percent depending on the method of re-contacting, with a higher response rate when contacting through the medical professionals compared to contacting the participants directly.

Apart from updating phenotypic data, additional measurements such as dynamometrics, electrocardiogram and spirometry have been taken as part of the re-contacting projects. Re-contacting provides an opportunity to take new samples (of different tissue or the same tissue). For instance, buccal swabs were obtained in addition to a second venous blood sample from the participants during their second visit. From that venous blood, RNA and serum were isolated for biochemical analysis. This makes it possible, for instance, to conduct studies on epigenetics as well as measure changes in metabolomics markers [5] at multiple time points.

Collecting new samples or data through re-contacting is limited and the whole process is time-consuming. In order to have a rich clinical characterization of a large part of the participants and update the database regularly, in a timely manner and at low cost, retrieving data from registries and other databases is much more efficient.

### 3.4. Data Updates from Registries and Hospital Databases

Estonia has a variety of national registries and databases with an extensive amount of collected data. As permitted by the HGRA, the EGCUT can take advantage of these existing infrastructures and retrieve participants’ data from these registries for phenotypic augmentation of the EGCUT cohort. This enables data on participants to be gathered retrospectively as well as the retrieval of information updates from registries and databases to continue on a regular basis. Overall, the EGCUT has retrieved data from nine different registries or databases (Figure 1). The aim is to retrieve data from these different sources annually, or according to the registry’s organization policies.

The first linking project was carried out in 2010 where the home addresses of the EGCUT participants were confirmed and/or updated from the Estonian Population Register in order to reach them for the re-contacting project (Figure 1). Linking with the Population Register has since been carried out on a regular basis a total of six times. Next, the EGCUT started linking annually with the Estonian Causes of Death Registry where for the past six years the date, cause and country of death of the diseased EGCUT participants are obtained.

Diagnoses, analyses and therapy information for specific disease classes are retrieved from the Estonian Cancer Registry, from the Estonian Tuberculosis Registry and from the Myocardial Infarction Registry.

Since 2013, the EGCUT started retrieving even more thorough health data from the hospital databases of Tartu University Hospital and the Northern Estonian Medical Centre, two of the main hospitals in the country. The information obtained includes the diagnoses, hospital discharge records, laboratory data, and imaging data from as far back as 1993. Overall, linkage with the two hospital databases has so far provided 320,000 unique confirmed diagnoses and up to 70,000 entries including 5000 clinical biomarker measurements. This introduced a completely new dimension in terms of the amount and detail of health information available on the biobank participants.

Additionally, data regarding the prescriptions provided and used, specialty and types of care provided as well as billing information is obtained from the Estonian Health Insurance Fund (HIF), that includes the Digital Prescription Database. The HIF contains information for all individuals covered by the national health insurance and the EGCUT has obtained over 3.2 M treatment documents and over 4.8 M diagnoses linked to the biobank participants in total for the period of 2003–2013 (Table 1). Linking with the Estonian National Health Information System (ENHIS) was carried out in 2014. Electronic health records (EHRs) and digital prescriptions from all medical service providers were retrieved from the ENHIS database including 44,000 inpatient and 212,000 outpatient medical summaries of the EGCUT participants.

**Table 1:** Proportion of Estonian Genome Center of the University of Tartu (EGCUT) participants’ represented per data source.

Data Source	No. of Participants Found *	Percentage of EGCUT Participants Represented
Population Register	51,800	99.8
Estonian Health Insurance Fund	51,607	99.5
ENHIS	39,880	76.9
Tartu University Hospital	22,492	43.4
North Estonia Medical Centre	21,202	40.9
Estonian Cancer Registry	2644	5.1
Estonian Causes of Death Registry	2349	4.5
Myocardial Infarction Registry	945	1.8
Estonian Tuberculosis Registry	260	0.5

* As per December 2014. ENHIS—Estonian National Health Information System.

### 3.5. Research Potential

The information available from all the registers in addition to the baseline data and biological samples adds immense value to the database, which translates into a variety of research possibilities. Information for the EGCUT population cohort includes genetic information, behavioral and lifestyle information, and health information. Through re-contacting and regular data retrieval from EHRs and national registries EGCUT possesses extensive follow-up data, including the confirmed diagnoses, clinical visits, laboratory analyses, treatment information as well as costs of health-care services that accompany the genetic information of participants.

**Figure 2:**
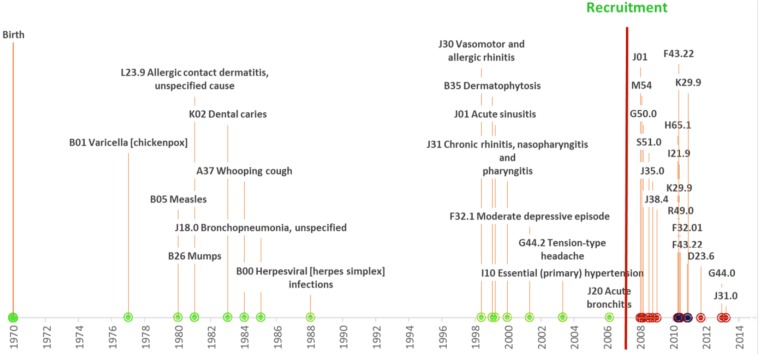
An individuals’ disease trajectory. A participant born in 1970 and recruited in 2007. Different data sources or collection methods are color coded; green—information gathered at the baseline interview, red—retrieved from Health Insurance Fund database, and blue—retrieved from the Northern Estonian Medical Center. The health status updates coded according to the ICD-10.

The phenotype data can periodically be updated. This results in a continually growing and up-to-date clinical characterization of the participants. Instead of just having a single time point with health history, EGCUT has longitudinal data with regularly added time points. Together with the Causes of Death Registry (as of 2014 mortality data are available for 2349 biobank participants) full disease trajectories can be studied (Figure 2).

This makes the EGCUT cohort and database an excellent source for conducting EHR-driven genomic research (EDGR) [6] and translational research including cost-effectiveness analysis. The relative properties of EDGR in comparison to conventional cohort studies where clinical characterization is obtained at recruitment and through re-contacting projects when possible have been described thoroughly by Kohane *et al.* [6].

## 4. Info-Technology, E-Solutions and Related Projects

### 4.1. National Infrastructure

Estonia has had several nationwide IT-development initiatives in the health sector that have also had a major impact on the growth of the EGCUT database as well as the potential to implement the results from genetic research into medical care. The key elements include the introduction of the national electronic identification card, the launch of the ENHIS system, as well as implementing digital prescriptions [7,8].

A nationwide governmental technical infrastructure, called X-road, allows secure communication between various registries and databases. This means that medical data from a hospital, primary care facilities, and pharmacies can all be accessed in a regulated manner. Secure e-services in Estonia are made possible through the use of a national electronic identification card (ID-card). The chipped national ID-card was implemented in 2002 and made compulsory. The digital signatures given with the ID-card are legally equivalent to a hand-written signature. The ID-card is used to access different services including the EHR both by the authorized medical personnel as well as the citizens.

Healthcare providers have been obliged to forward medical data electronically to the ENHIS since 2008. As of January 2015, the ENHIS contains over 14 million medical documents for over 1.3 million individuals in the database [7]. The system integrates data from various healthcare providers, who may use different systems, and generates a common electronic record for each person in a standardized format.

Projects like ENHIS ease the everyday work of health-care providers and improve patient services. Physicians are able to provide medical services based on a patient’s complete health information provided by ENHIS. Also, the efficiency of the services increases as a result. For instance, the digital prescription project was launched in 2010, and by 2013 over 95 percent of the prescriptions were being issued electronically through X-road. Patients are also more involved and informed, as they are able to access their own records through a Patient Portal, review their visits and prescriptions, as well as control who has accessed their EHRs. Patients can thereby be more actively involved in disease prevention. Additionally, since all stages are electronic from reception to prescription, medical statistics are more comprehensive and easier to use. Thus, it is possible to retrieve national statistics to identify systematic problems, and measure health trends.

### 4.2. Management of the Data Collection

Supplementing data in the EGCUT database from multiple sources introduces a variety of challenges for the information system used to manage the data. With each external data source, data security risks increase and additional security measures have to be introduced. There are more challenges to protecting a participant’s identity with more stakeholders involved in the process.

Retrieving additional health related information from a variety of different sources can provide overlapping information, a situation that needs to be resolved. However, all registries and databases have their unique advantages. For instance, ENHIS contains medical documents (epicrises) from throughout the country but the information available in ENHIS is not as thorough and complete as in the hospital databases. Therefore, potentially valuable data could be missed without retrieving data directly from the hospital databases, which contain much more detailed health information. Information received from the HIF, on the other hand, provides mainly financial reporting on related health-care services. National registries are valuable as they contain nationwide, high quality data that has already been systematically processed and is therefore easy to work with. Much of the information retrieved from the registries involves specific data fields in a structured format. The data received from ENHIS contains epicrises in free text format from where the necessary information needs to be extracted with data mining procedures.

The information system must track what source data is received and differentiate between time points. The data management tools need to be able to detect overlapping or conflicting data for a particular time-point. Additionally, data collection from different sources with different standards of practice introduces a challenge in terms of quality control. All these variables between augmented data to phenotype characterization of participants pose a challenge for fully understanding the discrepancy between details. The complexity of phenotypic characterization increases the challenges of data usability for research purposes. For research purposes management tools are necessary for flexible data inquiry where it is possible to make inquiries based on specific individuals, selecting specific time points or differentiating between the sources. Additionally, a systematic approach needs to be developed to evaluate reliability.

### 4.3. Future of the EGCUT Information System

The information system and IT solutions of the EGCUT need to continually evolve to support the goals of the EGCUT and the ever-increasing database. The main goals have shifted from recruitment to research supplementing and updating information on participants’ health. The HGRA and technical solutions available provide an opportunity to update the participant’s phenotype characterization from multiple sources over multiple time points.

Multiple data sources with varying standards of practice and different methods for describing medical information and data for many time points introduce a great challenge for data consolidation and harmonization. The system needs to be flexible and easily manageable in order to differentiate potentially overlapping or conflicting data, as well as to keep track of the different time points. To address these possibilities and challenges, the development of a new information system for the EGCUT, called GEVA (GEeniVAramu, which is short for EGCUT in Estonian), was initiated in 2012. GEVA is planned to support data collection from multiple sources by providing powerful tools for data consolidation from multiple sources and for quality control of data added to participants’ phenotype characterization.

Previously, data from registries and databases were retrieved through data packages after an application to the ministry in charge of the registry. Custom reports from the registry or database have been encrypted and handed over to EGCUT where they had to be manually added to the database. In a modern IT architecture, data will be exchanged securely between databases via X-road in real-time.

## 5. Translating Research Results to Medicine

Estonia is one of the countries aiming to implement personalized medicine on a national scale. Combining the IT infrastructure solutions available in Estonia with the comprehensive, longitudinally recorded electronic health records and prescription data, together with the genetic information available in the EGCUT, these resources are cornerstones for national personalized medicine initiatives. This information can be combined for clinical use in disease risk assessment and medication response prediction. In 2014, the Estonian Government allocated funds to establish a plan towards personalized medicine based on modern gene technology [9]. The pilot project for 2015–2018 was approved in December 2014 [10]. The plans include genotyping all 52,000 EGCUT participants on whom the personalized medicine approach can be pilot tested, and developing the necessary clinical decision support software. The ID-card and the patient portal of ENHIS allow the risk assessments to be delivered securely to physicians and patients.

The connection between the genomic research (EGCUT) and the health-care practice (ENHIS) makes it possible to gather evidence from routine clinical care, a method described as “learning health-care system” [11]. In a new and developing field such as genome-based medicine, a limited chain of events from data collection to clinical implementation to outcomes-research is necessary to learn and evolve in a timely manner.

## 6. Conclusions

The Estonian Biobank is a population-based biobank of the EGCUT with a collection of genetic and health data for a cohort of 52,000. After a period of recruiting participants from 2002 to 2010 the priorities shifted towards updating and enhancing the database for the cohort through re-contacting the participants as well as through retrieving data from national registries and hospital databases on a regular basis. This continuously growing and up-to-date database provides an excellent means for a variety of research possibilities. We described the elements that supported data collection through re-contacting or linking with registries and databases and mentioned some of the challenges that the EGCUT has faced during the process. The nationwide IT infrastructure in the health sector has not only been valuable for the growth of the database but will also play an important part when introducing the results from genetic research to healthcare practice as planned in the national personalized medicine pilot project in 2015–2018.
